# Internal Temperature Measurement of Optically Levitated Particles in Vacuum by Raman Thermometry

**DOI:** 10.3390/mi16121388

**Published:** 2025-12-07

**Authors:** Kou Li, Jiaming Liu, Xincai Xu, Zhuangzhuang Wang, Nan Li, Han Cai, Wenqiang Li, Huizhu Hu

**Affiliations:** 1 State Key Laboratory of Extreme Photonics and Instrumentation, College of Optical Science and Engineering, Zhejiang University, Hangzhou 310027, China; 22330016@zju.edu.cn (K.L.);; 2 Institute of Fundamental and Transdisciplinary Research, Zhejiang University, Hangzhou 310027, China

**Keywords:** optical levitation, Raman thermometry, photothermal effect, fused silica

## Abstract

An optical levitation system in a vacuum is an efficient system to investigate the dynamics of isolated micro- and nanoparticles. However, the motion and stability of the trapped particles in this system can be affected by the internal temperature, which remains a challenge to measure. Conventional methods are constrained by material specificity or lack the capability for direct temperature measurement. Here, we demonstrate the application of Raman thermometry for non-contact temperature detection of an optically levitated fused silica sphere in vacuum. In addition, the experimental results reveal a linear increase in particle temperature with laser power, consistent with photothermal theory. The integration of Raman thermometry with the optical levitation system enables high-precision thermal sensing at the microscale, offering significant potential for applications in precision metrology and fundamental physics.

## 1. Introduction

Optical levitation is a technology that can stably levitate micron- and nano-sized particles in air or vacuum. Since the pioneering proposal of optical levitation in 1970, this technique has evolved into a vital tool for precision measurement and exploration in modern physics. The levitated particle avoids contact with external mechanical elements, effectively isolating the measurements from clamping noise [1]. Optical levitation systems can enhance the signal-to-noise ratio and achieve highly sensitive detection. Consequently, this technology has great potential in high-sensitivity sensing and has become a vital physical platform for precision measurement, microscopic thermodynamics, and the exploration of macroscopic quantum states [2,3]. It also enables the accurate measurement of key physical properties such as mass [4] and geometry [5]. Over recent decades, this technology has gained much interest and has been widely researched and improved.

However, the internal temperature of the levitated particle remains a critical factor that significantly affects the performance and stability of these systems [6,7]. Thermally induced vibrations and the increased collision between the levitated particle and residual gas molecules accelerate the decoherence, directly decreasing the quantum coherence time of macroscopic quantum states. In addition, temperature gradients may intensify the phase instability and dissipation of energy, affecting the state stability and limiting the achievable force sensitivity in precision sensing [8]. Moreover, several studies have suggested that the thermal effects of the levitated particle may be responsible for their escape during the reduction in gas pressure [9,10]. In particular, the photoluminescence force induced by the internal temperature gradients may be a potential loss mechanism at pressures around 30 mbar [11].

To quantify the internal temperature of the levitated particle, several methods have been proposed, including luminescence thermometry by Yb^3+^:YLF nanocrystals [12,13], center-of-mass (COM) motion thermometry [14,15,16], displacement sensing [17], nitrogen vacancy thermometry [11,18], and Raman spectroscopy thermometry [19,20]. Luminescence thermometry by Yb^3+^:YLF nanocrystals has high sensitivity and non-contact operation at the nanoscale. However, this method requires doping rare-earth ions into the host nanoparticles, which may affect the stability of the particle in high vacuum. While center-of-mass motion thermometry has an excellent performance in high vacuum, it does not measure temperature directly. Instead, it works by analyzing the motion of the particle through a combined thermodynamic and kinetic model to infer its temperature. The requirement for multiple input parameters, however, introduces substantial uncertainty into the final temperature measurement. Meanwhile, nitrogen-vacancy thermometry offers high sensitivity and a wide range of adaptability to temperature, though its application is limited to diamond-based materials.

Among these approaches, Raman spectroscopy thermometry provides an in situ measurement capability for measuring the temperature of particles without contacting or disturbing the equipment operation or trapping stability of the sample. It determines temperature through universal molecular vibrations instead of the internal energy level, and thus, has broad material compatibility and high spatial resolution [21,22]. Raman spectroscopy thermometry enables non-contact thermal measurements with a resolution of ∼1 K while maintaining compatibility with ongoing optical manipulation [23]. Owing to these advantages, Raman spectroscopy thermometry has the potential to become an ideal tool for micrometer scale temperature analysis. It is also widely used in molecular structure analysis across chemistry, physics, materials science, and biomedicine [24,25,26,27,28].

In this paper, we developed an optical-levitating Raman thermometry system to investigate the thermal behavior of fused silica spheres under equilibrium conditions. Initially, we developed a vertical optical levitation system capable of capturing particles at the micrometer scale. Subsequently, a high numerical aperture (NA = 0.8) objective lens was utilized to ensure the collection of weak Raman signal scattered by the trapped particle. Prior to temperature measurements of the particle, we performed a separate calibration using a fused silica slice to establish the precise relationship between Raman shift and temperature. Ultimately, we achieved in situ temperature sensing of the levitated particle with an accuracy of ±5 K across varying air pressures and laser power levels.

## 2. Principle

Due to the momentum effect of photons, particles can be stably trapped in an optical levitation system. In the vertical optical levitation system, the trapped microsphere is primarily subject to optical trapping forces, molecular collision forces, air damping force, and gravity.

Molecular collisions with residual gas molecules introduce stochastic Brownian forces that drive random particle motion. For large objects, molecular collision forces on their surfaces largely cancel, resulting in negligible net force. However, for small particles, these collisions are typically imbalanced. Consequently, a particle may experience a strong impact in a particular direction at any moment, causing motion in that direction.

Air damping force is the frictional force experienced by a microsphere moving in air, with a magnitude proportional to the particle’s velocity.

Gravity is a constant force in a terrestrial environment, dependent on the object’s volume and density. For instance, an ideal spherical silica microsphere with a radius of 2.5 μm experiences a gravitational force of 1.44 × 10^−13^ N.

**Figure 1:**
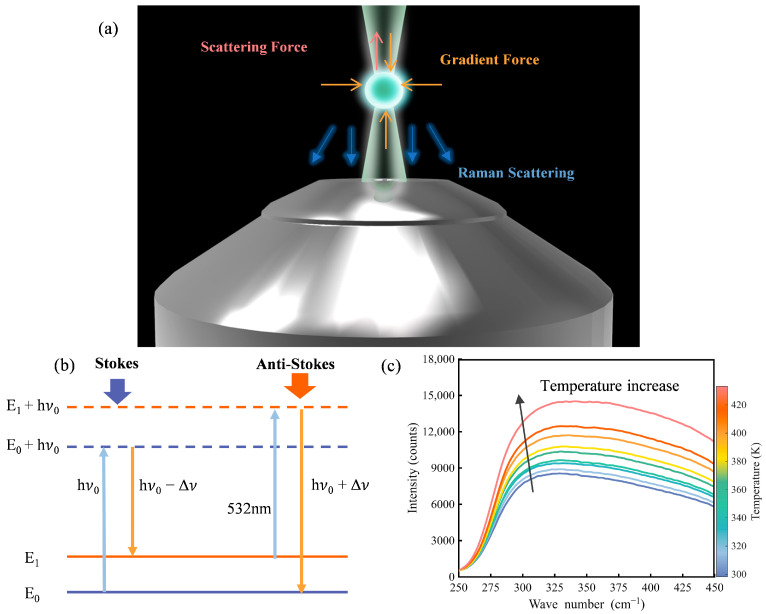
Overview of the experiment. (**a**) Schematic of a levitated sphere of fused silica, where the particle is captured by the beam and excited with Raman-scattered light; (**b**) energy level jump diagram of fused silica. Excitation at 532 nm is shown, producing shorter wave-length anti-Stokes light and longer wavelength Stokes light; (**c**) Raman spectra of a fused silica slice at different temperatures, with the arrow direction pointing to the temperature increase from 298 K (±1 K) to 433 K (±1 K). All spectra were acquired with a laser power of 150 mW and an integration time of 9 s. The plot focuses on the anti-Stokes partial region to clearly visualize the temperature-dependent spectral changes.

As shown in Figure 1a, a tightly focused beam along the optical axis (vertical) applies optical trapping forces on the microsphere, including both gradient and scattering forces [10,29]. The gradient force originates from the spatial intensity profile of the beam and draws the particle toward the focus, where intensity is highest. In contrast, the scattering force arises from photon momentum transfer along the propagation direction and tends to displace the particle axially. In vertical single-beam optical trapping, the optical trapping forces from the laser balances the weight of the microsphere, leading to zero net force along the vertical direction. Radially, the gradient force acts as a restoring force that centers the particle within the beam, enabling full three-dimensional confinement. This allows for three-dimensional confinement and stable trapping of the microsphere.

The optical levitation system provides an ideal platform for isolating and precisely manipulating individual particles.

In this study, a fused silica sphere is selected as the levitated particle owing to a combination of advantageous properties that make it highly suitable for optical levitation and subsequent precision measurements [30]. Its broad optical transparency—spanning from the deep ultraviolet to the infrared—ensures minimal light absorption at the typical trapping wavelength (e.g., 532 nm), thereby reducing laser-induced heating and preserving particle stability while lowering thermal noise in sensitive experiments. Furthermore, fused silica exhibits exceptional chemical inertness and mechanical robustness, allowing the particle to maintain its structural integrity and surface morphology over prolonged trapping durations, even under high-vacuum conditions. The material’s low impurity content (<1 ppm in synthetic grades) and high refractive index homogeneity further enhance its performance in optical levitation systems by minimizing unwanted scattering and absorption losses [31]. And fused silica spheres are commercially produced with excellent size uniformity and smooth surface finish, which facilitates accurate optical force modeling and reduces uncertainties in parameter extraction—critical aspects for achieving reliable mass and temperature measurements in optical levitation systems.

This capability is ideally complemented by Raman spectroscopy, which serves as a non-contact, highly sensitive method for probing the internal temperature of the particle without disturbing the trap.

Raman spectroscopy probes molecular vibrations and rotations by detecting frequency-shifted scattered light resulting from inelastic photon–matter interactions. When incident light interacts with a sample, most photons are elastically scattered (Rayleigh scattering), while a small fraction undergo inelastic scattering, resulting in an energy shift that corresponds to the vibrational modes of the material [32], as shown in Figure 1b. This change in the frequency of the scattered light (i.e., the Raman shift) correlates with the vibrational mode of the sample.

Raman spectroscopy exhibits thermal dependency [33]. Specifically, the polarizability of matter, which governs Raman scattering, is highly sensitive to temperature. These changes manifest as measurable shifts in Raman spectral features, including peak position, linewidth, and the relative intensity between Stokes and anti-Stokes bands. As illustrated in Figure 1c, we observe that the anti-Stokes signals change regularly during the temperature rise in a fused silica slice. Consequently, Raman signals are suitable for temperature detection and heat transfer analysis.

Conventional methods include frequency-based, linewidth-based, and intensity-based thermometry [34]. The frequency-shift method offers high precision and is well-suited for steady-state temperature fields, while the linewidth-based approach exhibits good stability in high-temperature regions and is often used as a complementary technique. As Raman intensity thermometry requires less signal strength and is more widely applicable than the other two methods, intensity-based thermometry is the most prevalent Raman thermometry technique and was selected for this study.

The intensity ratio of the anti-Stokes to Stokes spectra is closely related to temperature. If the incident frequency is $\omega_{L}$, the scattering cross-sections for Stokes and anti-Stokes are:

(1) $${\left(\frac{d\sigma }{d\Omega }\right)}_{Stokes}={r_{0}}^{2}\left(\frac{\omega_{S}}{\omega_{L}}\right)\left(n_{S}+1\right){\left|\hat{e_{S}}\cdot \tilde{R}\cdot \hat{e_{L}}\right|}^{2}$$

(2) $${\left(\frac{d\sigma }{d\Omega }\right)}_{antistokes}={r_{0}}^{2}\left(\frac{\omega_{A}}{\omega_{L}}\right)\left(n_{A}+1\right){\left|\hat{e_{A}}\cdot \tilde{R}\cdot \hat{e_{L}}\right|}^{2}$$

where $\tilde{R}$ is the Raman tensor, $r_{0}$ is the classical electron radius, $\omega_{S}$ and $\omega_{A}$ are the frequency of Stokes and anti-Stokes, $\hat{e_{L}}$ denotes the unit polarization vector of the incident laser field, $\hat{e_{S}}$ and $\hat{e_{A}}$ are the unit polarization vectors of the scattered Stokes and anti-Stokes light, respectively. And the Stokes ($n_{S}$) and anti-Stokes ($n_{A}$) photon populations follow the Boltzmann distribution:

(3) nS+1nA+1≅nSnA=e−E0kTe−E1kT=eΔEkT

where *k* is the Boltzmann constant, *T* is the temperature and $\Delta E=E_{1}-E_{0}$ is the energy difference between the vibrational energy levels involved in the Raman process, as shown in Figure 1b. Here, $E_{0}$ denotes the ground-state energy of the molecule and $E_{1}$ represents the energy of the vibrationally excited state.

Considering photon modes, the photon state number (mode number) $g_{\Delta \nu }$ within frequency interval Δν and solid angle ΔΩ is:

(4) $$g_{\Delta \nu }=\frac{\Delta \Omega }{4\pi }\frac{8\pi \nu^{2}\Delta \nu }{c^{3}}V=\frac{2\nu^{2}\Delta \nu }{c^{3}}V\Delta \Omega$$

where c is speed of light.

According to the Boltzmann distribution governing the vibrational energy level populations under thermal equilibrium, the relationship between the Stokes and anti-Stokes signal intensity ratio is [35]:

(5) ISIAS=νl−νpνl+νp3γehνpkTint

Here, $I_{S}$ and $I_{AS}$ are the intensities of the Stokes and anti-Stokes signals, *ν_l_* is the incident laser frequency, *ν_p_* is the phonon frequency corresponding to the Raman shift, *k* is the Boltzmann constant, *T_int_* is the internal temperature, and h is the Planck constant. *γ* is the correction factor including the differential detection efficiency and optical transmission at the Stokes and anti-Stokes wavelengths. Under stable experimental conditions, it is treated as a constant and its value is determined by calibrating the intensity ratio at known temperatures.

## 3. Experiment and Results

**Figure 2:**
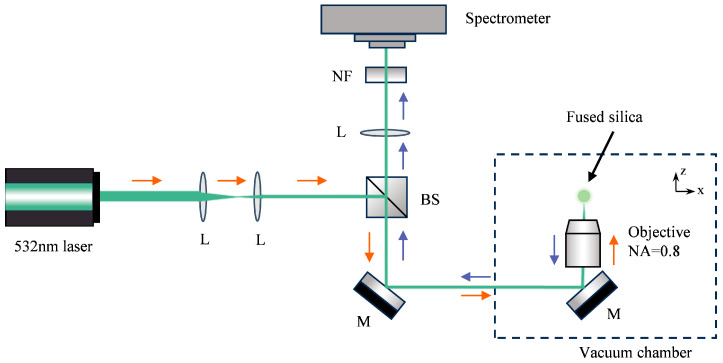
Experimental setup for in situ Raman thermometry (M, reflective mirror; BS, beam splitter; L, lens; NF, notch filter). A 532 nm laser (orange arrows) is focused to trap a single fused silica microsphere. The trap is placed in a vacuum chamber to adjust the gas pressure. The backscattered Raman light (blue arrows) from the particle is collected and directed to a spectrometer.

The experimental setup is illustrated in Figure 2. The experiment was performed within a vacuum chamber capable of pressure regulation spanning from atmospheric pressure down to approximately 10^−3^ mbar. A trapping laser (λ = 532 nm) was focused through a high-numerical-aperture objective (NA = 0.8). A fused silica sphere (diameter = 5 μm) was loaded into the optical trapping region from a vibrating glass slide driven by piezoelectric transducer (PZT). The Raman signal was generated within the optically levitated particle under laser excitation and collected in a backscattering geometry by the same objective. The backscattered Raman signal was directed through a beam splitter (BS) and subsequently passed through a notch filter (NF) to suppress the 532 nm laser line. The filtered Raman signal was then delivered to the spectrometer (ANDOR SR-750) for detection. And the spectrometer was equipped with an EMCCD detector (ANDOR Newton DU970N-BV) capable of single-photon detection for signal acquisition. The Stokes and anti-Stokes spectra were acquired separately by configuring the spectrometer to the respective spectral regions in sequence.

Using the optical levitation device described above, we investigated the internal temperature of the particle. In this study, we work with air pressures ranging from 20 mbar to 200 mbar, where particles undergo collisions with gas molecules. To extract quantitative temperature from the Raman spectra, a precise calibration of the relationship between the Stokes/anti-Stokes intensity ratio $I_{S}/I_{AS}$ and temperature is essential, especially due to the system-dependent correction factor *γ* in Equation (5). This can be accomplished through experimental calibration by measuring the Raman intensity ratio at known temperatures to construct a calibration curve.

A fused silica slice was fabricated from the same material as the levitated spheres. With its known, controllable temperature and enhanced signal, this slice was suitable for utilization in the precise calibration. To ensure the validity and consistency of the calibration, all measurements were conducted on the same system as in the trapping experiments, using the same objective and optical path. The temperature control up to 473 K (±1 K) can be achieved using the heating stage. In our experiment, for a given fused silica slice, the temperature of the heating table is set from room temperature to 433 K (±1 K). After the temperature measured by the electronic thermometer is basically stable, the actual temperature of the fused silica slice is recorded and the Raman spectral data is measured.

**Figure 3:**
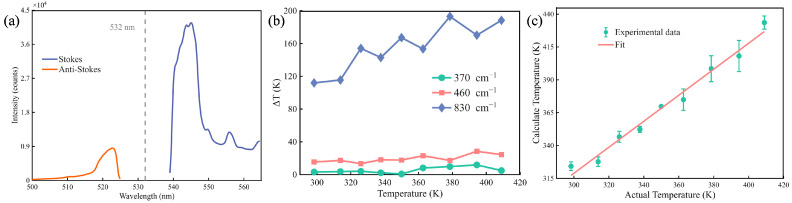
Calibration results of Raman thermometry. (**a**) The smoothed data clearly reveal the characteristic peaks of the Stokes and anti-Stokes lines. The Stokes line, indicated by the blue curve, appears at a higher wavelength compared to the anti-Stokes line (orange curve), consistent with the typical Raman scattering pattern. The dashed line marks the 532 nm laser excitation wavelength, providing a reference for the spectral shifts. The spectra of Stokes and anti-Stokes were separately acquired with a laser power of 150 mW and an integration time of 9 s. (**b**) Temperature errors (ΔT) obtained from different wave numbers at the temperature from 300 K to 410 K were calculated. Here, ΔT is defined as the maximum deviation from the mean among these measurements. (**c**) The relationship between the calculated internal temperature of the fused silica slice by Raman spectroscopy at 370 cm^−1^ and the actual temperature measured by an electronic thermometer at room temperature (298 K).

After smoothing the collected Raman spectra data, the signal results are shown in Figure 3a. The Stokes peak is more intense than the anti-Stokes peak due to the higher population of lower vibrational energy states at the measurement temperature. The Stokes and anti-Stokes bands were then symmetrically processed to ensure consistent line shape analysis. This result was calculated using Equation (5) to obtain the calculated temperature at each spectral point. And the final calculated temperature was determined by averaging these point-by-point values across the characteristic Raman peak in 10 cm^−1^ windows. The analysis focused on the three main and representative characteristic peaks of fused silica centered at 370 cm^−1^, 460 cm^−1^, and 830 cm^−1^. Figure 3b shows the relationship between actual temperature ($T_{actual}$) and temperature errors (Δ*T*) for different Raman shifts. Here, Δ*T* is defined as the maximum deviation from the mean among these measurements. Δ*T* is slightly larger at 460 cm^−1^ and increases significantly with temperature at 830 cm^−1^. In contrast, at 370 cm^−1^, Δ*T* remains small (±5 K) and stable across the measured temperature range, as 370 cm^−1^ is the most intense peak in the spectrum [28]. This indicates minimal deviation between measured and actual temperatures, and higher measurement accuracy and stability during measurement. We selected 370 cm^−1^ for temperature calibration and subsequent measurements.

The actual and calculated temperatures is calibrated with data at 370 cm^−1^, as shown in Figure 3c. It indicates a linear relationship between calculate temperature and actual temperature, which is in great agreement with the theoretical predictions derived from the Boltzmann relation between the Raman scattering intensity ratio and the temperature of fused silica. And the relationship is shown:

(6) $$T_{actual}=\beta \times T_{calculated}+T_{offset}$$

Here, β=1.010 represents a scaling factor and $T_{offset}=-22\;K$ is a systematic temperature offset.

**Figure 4:**
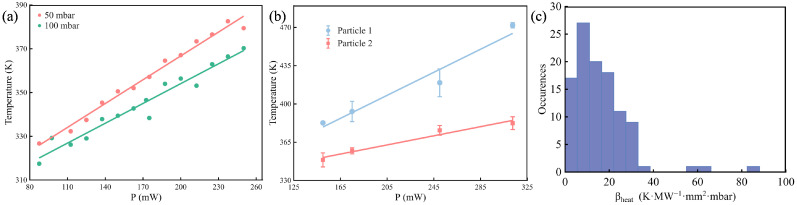
Heating experiments performed on fused silica particles. (**a**) Typical recordings of the internal temperature of the fused silica particle as a function of laser power density at different gas pressures: 50 mbar (red) and 100 mbar (green). (**b**) The temperature of two different particles at 50 mbar. Both exhibit a rising temperature trend as laser power increases, but Particle 2 consistently shows a higher temperature than Particle 1 at the same power levels. (**c**) Histogram of heating coefficients $\beta_{heat}$ recorded on more than 50 fused silica spheres reflecting differences in photothermal conversion of different fused silica spheres. The distribution is skewed to the right, with most spheres having $\beta_{heat}$ values on the lower side, but some exhibiting much higher values.

Based on the calibration results, we further investigated the internal temperature of the levitated particle. Figure 4a shows the relationship between laser power (P) and particle temperature (T) under two different pressure conditions (50 mbar and 100 mbar). The laser power was measured at the transmission port of the beam splitter (BS) and is identified as the optical power incident on the levitated particle. Temperature increases linearly with laser power, consistent with the photothermal effect theory, wherein the particles absorb laser energy and convert it into thermal energy, leading to a temperature rise. Compared to the 50 mbar condition, the rate of temperature increase is lower at 100 mbar. This reduction may be attributed to the increased density of gas molecules at higher pressure, which enhances heat exchange with the levitated particles. As a result, more heat is dissipated from the particles, leading to a slower temperature rise. These results indicate that the temperature of the particles can be effectively controlled by adjusting the laser power and this process is significantly affected by the ambient air pressure.

In addition, fused silica spheres always escaped from the trap at a pressure of about 10 mbar, possibly caused by an increase in internal temperature. And it is primarily related to the photophoretic force, which arises from temperature gradients within the particle and becomes significant when the gas mean free path is comparable to the particle size. Additionally, reduced gas damping at this pressure amplifies the effect of any directional perturbation. Therefore, we kept the fused silica spheres in their stabilized region, i.e., the control gas pressure was higher than 20 mbar.

However, we also observed particles with large temperature excursions. Figure 4b shows variations in particle temperature under identical conditions (50 mbar), with particle temperatures up to 470 K. In fact, the internal temperature of levitated particles arises from the competition between different thermal processes. According to the photothermal theory, the final temperature of the particle corresponds to the balance between the absorbed power $P_{abs}$ and the gas conduction $P_{cond}$ [18].

To characterize the absorption of particles, we introduce the absorption cross-section $\sigma_{abs}$, and

(7) $$P_{abs}=\sigma_{abs}(\lambda )I_{las}$$

where $I_{las}$ is the intensity of trapping laser.

The thermal power dissipated by gas conduction in the free molecular state (this condition was verified for the pressures studied) is [36]:

(8) $$P_{cond}=\alpha_{acc}S_{part}\frac{p_{gas}\overset{¯}{c}}{8T_{0}}\frac{\gamma +1}{\gamma -1}(T_{int}-T_{0})$$

where T_0_ = 298 K and $T_{int}$ are the ambient and internal particle temperatures, respectively; c = 503 m/s is the average thermal velocity of the gas molecules; $S_{part}$ is the particle surface area; *γ* = 7/5 is the gas ratio; and $\alpha_{acc}$ is the particle accommodation coefficient.

At thermal equilibrium, $P_{abs}=P_{cond}$, so the particle temperature is:

(9) $$T_{int}=T_{0}+\beta_{heat}\frac{I_{las}}{p_{gas}}$$

where we characterized the heating coefficient of a particular fused silica sphere in terms of $\beta_{heat}$:

(10) $$\beta_{heat}=\frac{\sigma_{abs}(\lambda )}{\alpha_{acc}S_{part}}\frac{8T_{0}}{p_{gas}\overset{¯}{c}}\frac{\gamma -1}{\gamma +1}$$

To further investigate the heating mechanism of fused silica spheres, we measured the heating coefficient $\beta_{heat}$ for more than 50 individual spheres. The distribution of $\beta_{heat}$ values is shown in Figure 4c. The data shows most heating coefficients fall around 15 K·MW^−1^·mm^−2^·mbar, meaning the silica spheres usually heat up very little under laser light, which can be attributed to their inherently low optical absorption and excellent thermal stability. This is useful for experiments where temperature must be kept steady. At the same time, a few particles heat up more easily, showing higher $\beta_{heat}$ values. These differences may come from small impurities or uneven structures inside the spheres. Such flaws can make the material absorb more laser energy, raising the heating effect.

## 4. Conclusions

This study investigates the principles of Raman thermometry and its application to measure the temperature of the optically levitated particle in vacuum. Optical levitation system employs a highly focused laser beam to capture and manipulate microparticles in vacuum. Raman spectroscopy probes the internal temperature of the trapped particle through the inelastically scattering process. The combination of these two techniques allows high-precision temperature measurement of the levitated particle in vacuum, which is considerably important for fundamental physics and precision metrology.

Based on Raman thermometry, our work investigates the influence of laser power on particle temperature at different pressure levels and analyzes the distribution of particle heating coefficients. The low heating characteristics of most fused silica spheres in our research have been found, making them well-suited for optical levitation applications. These results not only advance the understanding of optical levitation and Raman based temperature sensing mechanism but also provide the guidance for the design and implementation of precision measurement with optical levitation system. In future, we aim to determine the escape temperature of levitated particles. This will enable the use of Raman thermometry for in situ monitoring of particle stability.

## Data Availability

Data will be made available on request.
